# Inter hospital transfer or centralized ambulance number – what is the need of hour?

**DOI:** 10.1186/1865-1380-8-S1-P2

**Published:** 2015-04-22

**Authors:** Kaveri Bharati, Taraknath Taraphdhar, Sunil Choudhary

**Affiliations:** 1 The Mission Hospital, Durgapur, West Bengal, India

## Objective

Pre-hospital patient management with resuscitative procedures is a standard of care in mature EMS systems. Currently, no centralized EMS system exists for the population in Eastern India. This leads to patients presenting to under-resourced facilities highlighting the need for sophisticated ambulance services for inter-facility transfers. This study identifies the need of a centralized process to obtain ambulance services for timely transport of critically ill patients to adequately equipped tertiary care centers, thereby improving patient outcome and achieving the concept of golden hour.

## Methods

A retrospective observational review was done from January 2011 to December 2013 of The Mission Hospital ACLS inter facility transfer ambulance. All triage priority one patients being transferred to the emergency department were included. Demographic analysis was done by multivariate statistical methods.

## Results

1298 patients were transferred by ambulance during the study period, of which 462 patients (35.59%) belonged to triage category 1 during initial assessment. 449 patients (97.18%) were above 18 years of age, males being 257 (57.23%) and females being 192 (42.76%). Resuscitative efforts were performed on 236 patients (51.08%) including intubation in 58 patients (24.27%) of which 36 patients (62.07%) underwent RSI, with 41 patients (17.15%) being brought on ventilator, 6 patients (0.025%) on pacing and 131 patients (55.51%) received inotropes to manage cardiac instability. 3 patients expired before reaching the hospital.

## Conclusion

This study highlights the need to for the development of a centralized ambulance coordination service with appropriately trained personnel. This system would reduce the requirement of interfacility transports. This data shows that inter-facility ambulances in Eastern India were successful in transferring critically ill patients, but this situation has evolved due to the lack of a centralized EMS system and placing undue burdens on a few institutions.

